# Pulmonary amyloidosis, gastric MALT lymphoma, and multiple sclerosis in a patient with Sjögren’s disease: a case report and review of the literature

**DOI:** 10.1007/s00296-026-06276-0

**Published:** 2026-08-25

**Authors:** Nikolaos Zintziovas, Georgios A. Drosos, Melina Yerolatsite, Anna Goussia, Vasiliki Kostadima, Anastasia K. Zikou, Paraskevi V. Voulgari

**Affiliations:** 1 Department of Rheumatology, School of Health Sciences, University of Ioannina, 45110 Ioannina, Greece; 2 Medical School, University of Nicosia, Nicosia, Cyprus; 3 Department of Medical Oncology, University Hospital of Ioannina, Ioannina, Greece; 4 Department of Pathology School of Health Sciences, Faculty of Medicine, University of Ioannina, Ioannina, Greece; 5 Department of Neurology, University Hospital of Ioannina, Ioannina, Greece; 6 Department of Radiology, Medical School, University of Ioannina, Ioannina, Greece

**Keywords:** Sjogren’s syndrome, Lymphoma, Lymphoma, B-cell, Marginal zone, Multiple sclerosis, Amyloidosis

## Abstract

Sjögren’s disease (SjD) is a systemic autoimmune disorder associated with lymphocytic gland infiltration and an increased risk of B-cell lymphomas, particularly mucosa-associated lymphoid tissue (MALT) lymphoma. Neurological involvement may mimic multiple sclerosis (MS), while true coexistence, especially with amyloidosis, is exceptionally rare. We performed a systematic review and present a representative clinical case. A registered systematic literature review (PROSPERO CRD4201332325) was conducted according to PRISMA 2020. PubMed/MEDLINE, Scopus, the Cochrane Library, and DOAJ were searched for all records available up to July 2026. As no eligible cases were identified, additional targeted searches were performed to identify reports describing each possible combination of three of the four conditions. We also present a case report prepared according to the CARE guidelines. Among the 2,966 records identified, no studies describing the coexistence of Sjögren’s disease, amyloidosis, MALT lymphoma, and MS in the same patient were found. Additional targeted searches identified 14 case reports and one case series describing Sjögren’s disease with amyloidosis and MALT lymphoma, whereas no reports of Sjögren’s disease with amyloidosis and MS or with MALT lymphoma and MS were identified. We therefore report the first case of Sjögren’s disease complicated by pulmonary amyloidosis, gastric MALT lymphoma, and MS, highlighting the diagnostic and therapeutic challenges of this rare clinical presentation. The coexistence of Sjögren’s disease, pulmonary amyloidosis, gastric MALT lymphoma, and MS is likely to be rare. Although partial associations have been reported, our literature review did not identify any previous case describing all four conditions in the same patient. This case underscores the importance of a multidisciplinary approach and suggests that possible shared immunopathogenic mechanisms warrant further investigation. *Registration* PROSPERO CRD4201332325.

**Graphical abstract:**

**Figure:**
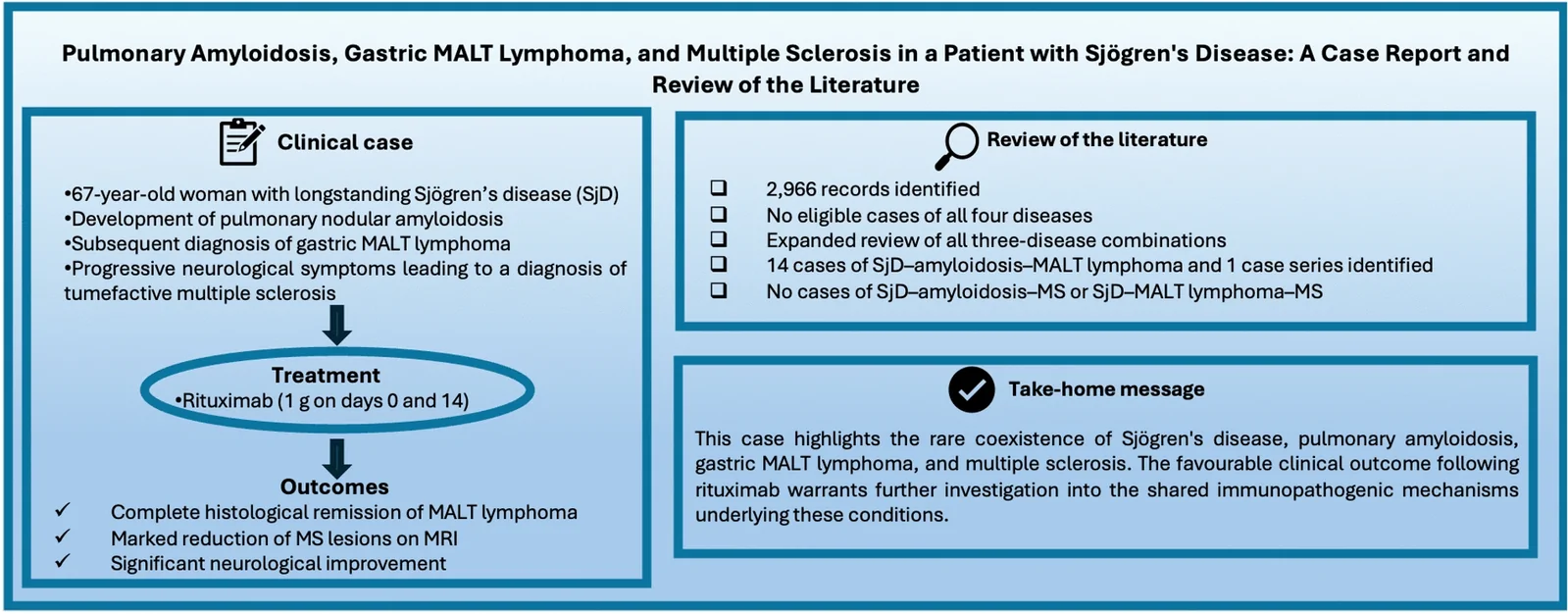

**Supplementary Information:**

The online version contains supplementary material available at https://doi.org/10.1007/s00296-026-06276-0.

## Introduction

Sjögren’s disease (SjD) is a chronic systemic autoimmune disorder characterized by lymphocytic infiltration of exocrine glands, leading to sicca symptoms and a distinctive predilection for lymphomagenesis, particularly mucosa-associated lymphoid tissue (MALT) lymphoma. Beyond exocrine gland involvement, SjD may affect multiple organ systems, including the lungs and the central nervous system (CNS) [1–3].

Specifically, SjD is associated with various pulmonary manifestations, including interstitial pneumonia, tracheobronchial disorders, and lymphoproliferative disease, whereas pulmonary amyloidosis is uncommon [4]. Amyloidosis comprises a heterogeneous group of rare disorders characterized by protein misfolding and subsequent tissue deposition, leading to organ dysfunction [5]. The majority of respiratory tract amyloidosis cases involve AL-type protein deposition [4].

Pulmonary amyloidosis is classified into three clinicopathologic subtypes: diffuse alveolo-septal, tracheobronchial, and nodular pulmonary amyloidosis [6]. Nodular pulmonary amyloidosis typically presents as incidentally discovered peripheral or subpleural nodules with an indolent clinical course. Radiological differentiation from lymphoma or primary lung malignancy is often challenging, necessitating invasive diagnostic procedures for definitive diagnosis. Most patients remain asymptomatic and require only radiological surveillance, while localized resection is generally curative. Importantly, accumulating evidence suggests that nodular pulmonary amyloidosis is frequently associated with underlying lymphoproliferative disorders, particularly SjD and extranodal marginal zone lymphoma (MZL) of MALT type [6–8].

Neurological involvement in SjD is relatively common and has been reported in approximately 10–20% of patients [9–13]. CNS manifestations may include demyelinating lesions resembling multiple sclerosis (MS), with hyperintense white matter lesions on T2-weighted and FLAIR Magnetic Resonance Imaging (MRI). Cerebrospinal fluid (CSF) abnormalities, such as an elevated IgG index and oligoclonal bands, may also be present in a subset of patients, further complicating the differential diagnosis with MS [9–13]. MS is a chronic inflammatory demyelinating disease of the CNS driven by autoimmune responses against myelin antigens [14]. MS shares several genetic and immunopathogenic features with SjD [15].

Distinguishing primary MS from neurological manifestations of SjD remains challenging, as patients with SjD may fulfill some or all diagnostic criteria for MS, including clinical demyelinating episodes, radiological dissemination in space and time, and supportive cerebrospinal fluid findings [9, 11, 16–18]. Sicca symptoms may occasionally be observed in MS, particularly in progressive disease forms, further underscoring the diagnostic overlap between the two conditions [15]. Although SjD and MS are generally regarded as distinct clinical entities, true overlap has been documented in several case reports and small series [5, 19, 20].

The true prevalence of SjD–MS overlap remains uncertain due to heterogeneous diagnostic criteria and limited cohort sizes. Available epidemiological data suggest that overlap is uncommon, with most studies reporting SjD prevalence among patients with MS ranging from 0% to 3.3%, comparable to that of the general population [12, 21–24]. In the largest multicenter study to date, Annunziata et al. reported a SjD prevalence of 0.91% among patients with MS, with subsequent studies confirming similarly low rates [6, 25].

Among patients with SjD, non-Hodgkin B-cell lymphomas are the most frequently encountered malignancies, with MZL representing the predominant subtype and typically arising within organs targeted by the autoimmune process, such as the salivary and lacrimal glands [2, 26].

Although the overlap between SjD and MS has been documented, the coexistence of these autoimmune conditions alongside gastric MALT lymphoma and amyloidosis is exceedingly uncommon. This study presents a systematic review accompanied by a narrative synthesis to examine the available evidence on the associations between SjD, amyloidosis, gastric MALT lymphoma, and MS, contextualized by a clinical case from our department. As no reports describing the coexistence of all four conditions were found, we expanded the literature review to include all possible combinations of three of the four diseases, providing a broader context for the present case.

### Case presentation

The present case is reported in accordance with the CARE (CAse REport) guidelines for case reporting [27].

### Patient information

A 67-year-old woman with a 30-year history of SjD, diagnosed by a positive minor salivary gland biopsy and seropositivity for antinuclear antibodies (ANA; titre 1/640, fine speckled pattern), anti-Ro60 (60 kDa) and anti-Ro52 (52 kDa) autoantibodies, was referred to the rheumatology clinic for further evaluation. The patient was receiving hydroxychloroquine for mild arthralgias, while the rest of her medical history included dyslipidemia, depression, osteoporosis, and stroke.

### Clinical history

#### Previous neurological history

Stroke was diagnosed 3 years before presentation, during the onset of blurred vision, vertigo with unstable gait and tremor. She underwent a brain computed tomography (CT) scan which revealed a low-density lesion on the right occipital lobe and some older microinfarcts of the white matter. Subsequent brain MRI revealed brain atrophy and microvascular encephalopathy, as well as some additional chronic subcortical infarcts on the right insular cortex. Further investigation revealed that she was also heterozygous for MTHFR variant and had patent foramen ovale. Since then, the patient has been on acetylsalicylic acid and statin treatment.

#### Pulmonary nodular amyloidosis

**Fig. 1:**
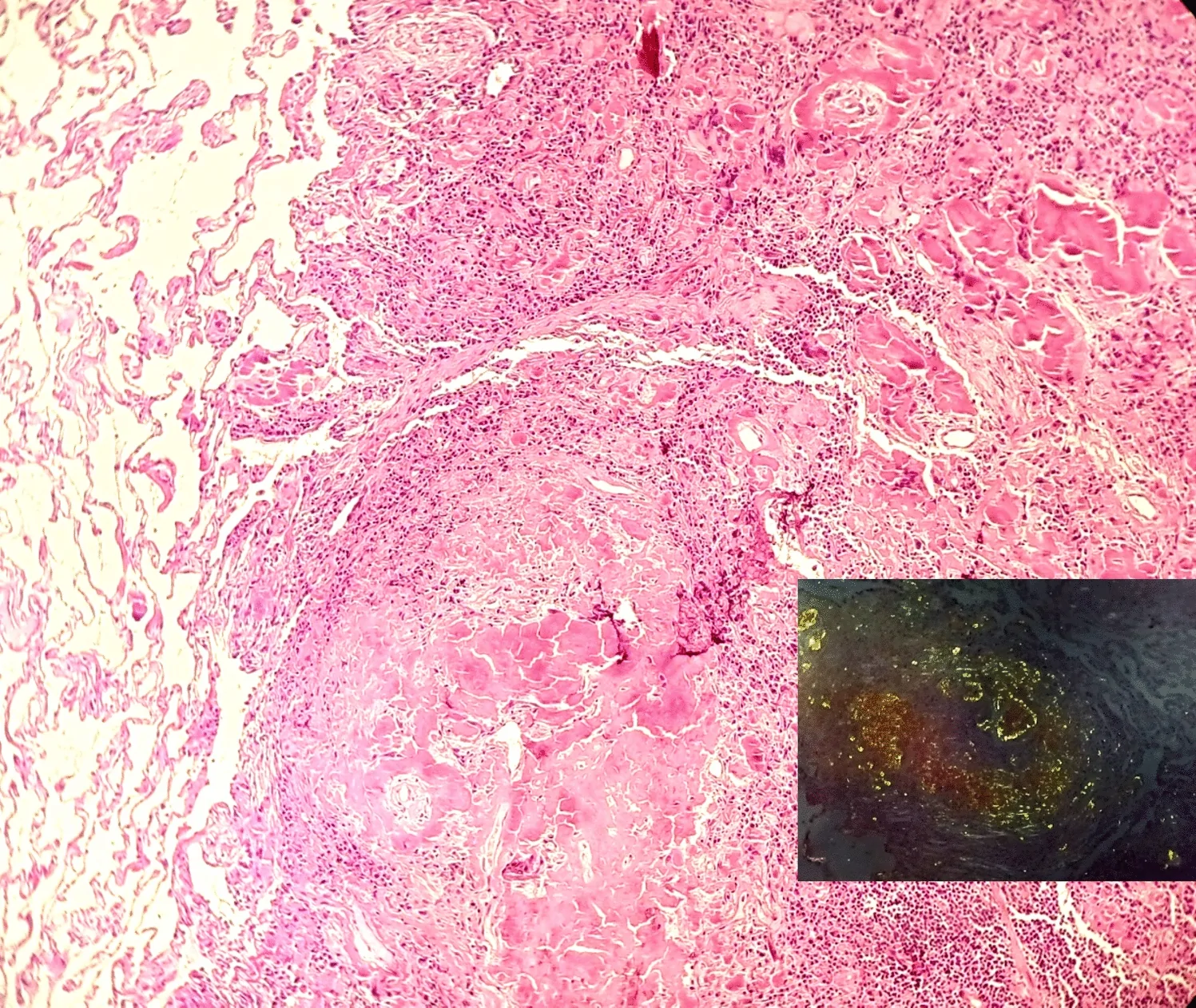
Lung parenchyma with extensive deposits of pink amorphous material within the interstitial tissue and vessel walls (Hematoxylin & Eosin stains, original magnification X40); the latter shows apple green birefringence with Congo red stain under polarized light (insert)

At about the same time, following the incidental discovery of pulmonary nodules in the left lung apex, the patient underwent a positron emission tomography–computed tomography (PET/CT) scan, which revealed metabolically active nodules, prompting surgical excision. Histopathological examination of the excised tissue demonstrated amyloid deposition, which was confirmed by Congo red staining under polarized light (Fig. 1). Amyloid typing was not performed. Based on these findings, a diagnosis of pulmonary amyloidosis associated with SjD was established.

#### Initial gastric evaluation

Nine months prior to the current presentation, the patient underwent gastroscopy for mild dyspepsia, which revealed mild gastritis. Biopsy specimens demonstrated infiltration by monoclonal cytoplasmic immunoglobulin/λ+ (CΙg/λ +) plasma cells as well as some small B cells. These findings raised the possibility of MZL with CΙg/λ+ plasma cell differentiation in the context of SjD. Although amyloid deposition was not confirmed, the possibility of plasma cell dyscrasia associated with amyloidosis could not be excluded. The patient received eradication therapy for Helicobacter pylori (H. pylori) and was referred to the hematology clinic, where she underwent bone marrow aspiration and biopsy. Bone marrow aspiration revealed a hypercellular bone marrow (BM) featuring all the cell lines, granulocytic and megakaryocytic hyperplasia, elements of red cell and megakaryocytic dysplasia and absence of increased blasts. The bone marrow biopsy demonstrated mild hyperplasia of hematopoietic cells in all stages of maturation, with 10% lymphocytic infiltration -predominantly T cells- and 5% polyclonal plasma cells. Congo red stain was negative for amyloid deposition. The complement levels were within normal limits, there were no detectable cryoglobulins, the blood flow cytometry was normal with a CD4+/CD8 ratio value of 1.05, and serum and urine protein electrophoresis did not reveal the presence of monoclonal globulin. Overall, hematological staging, including bone marrow aspiration and biopsy, peripheral blood flow cytometry, and serum and urine protein electrophoresis, revealed no evidence of systemic lymphoproliferative disease or plasma cell dyscrasia. In addition, contrast-enhanced CT of the chest and abdomen and 18F-FDG PET/CT showed no evidence of systemic disease.

#### Evaluation for systemic amyloidosis

For further systemic amyloidosis evaluation an abdominal adipose tissue biopsy was performed and found to be negative for amyloid deposition. Cardiac single photon emission computed tomography** (**SPECT) for the detection of myocardial amyloidosis was also negative, excluding myocardial amyloidosis. Based on these findings, a diagnosis of localized pulmonary nodular amyloidosis was confirmed.

### Diagnostic assessment

#### Neurological progression

In the months following her initial presentation to our clinic, the patient experienced progressive worsening of her neurological symptoms. A repeat brain MRI revealed an increase in the number and size of cerebral lesions compared to the imaging performed 3 years earlier. These lesions demonstrated diffusion restriction and mild peripheral enhancement following intravenous contrast injection and were considered to be of ischemic cause.

**Fig. 2:**
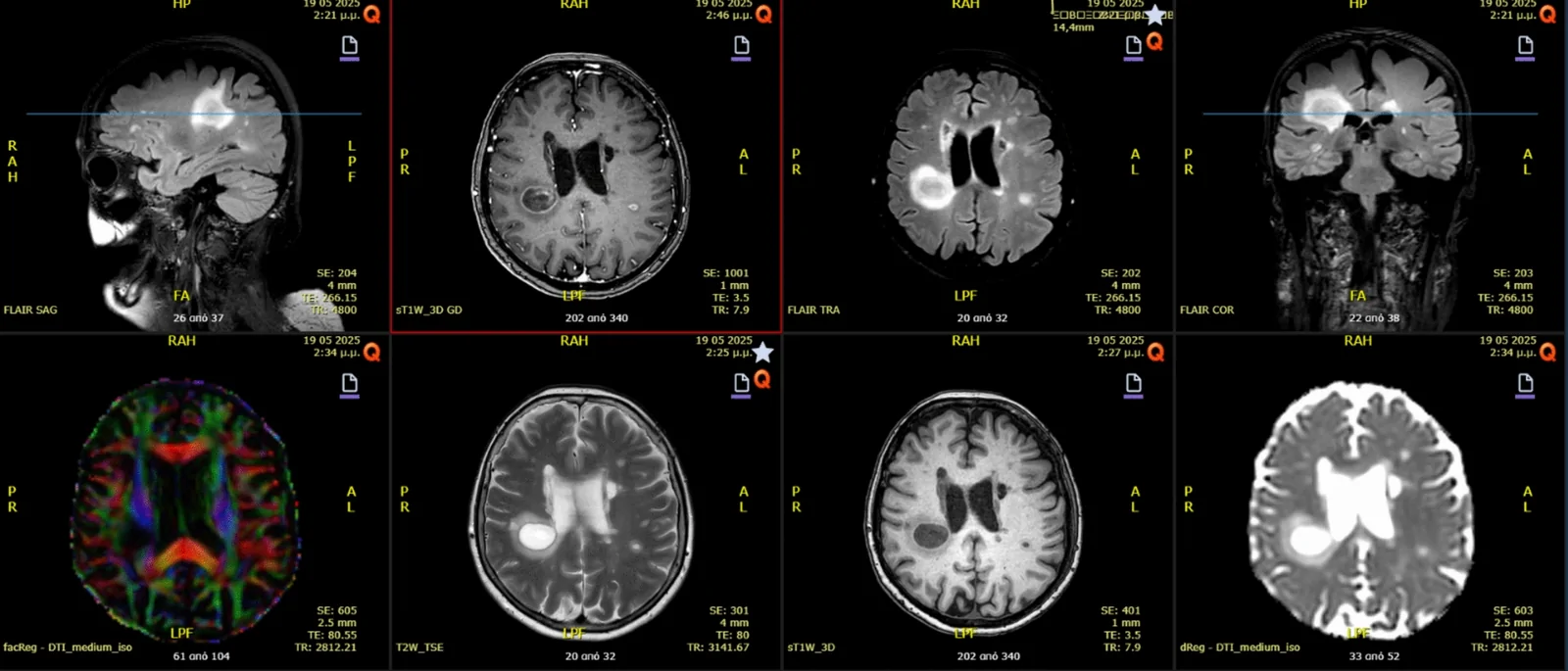
Brain MRI demonstrating tumefactive MS – Multiple hyperintense lesions on T2/FLAIR sequences with corresponding T1 hypointensity are visible in the periventricular and subcortical white matter. The dominant right parietal lesion (2.5 × 1.7 cm) exhibits a characteristic "double ring" appearance on FLAIR, with both peripheral and internal rings demonstrating enhancement following gadolinium administration. The lesion shows free diffusion on ADC maps with associated diffusion restriction in the enhancing rings. Perfusion imaging reveals no increased cerebral blood flow

Due to further clinical deterioration over the ensuing months, with worsening of gait instability, repeat brain MRI (approximately 6 months after the previous study) demonstrated multiple supra- and infratentorial lesions that were hyperintense on T2/FLAIR and hypointense on T1 sequences. Τwo lesions were located in the right cerebellar hemisphere, two in the pons, a third larger lesion, in the left cerebellar peduncle, and various lesions in the periventricular and subcortical white matter. A dominant right parietal lesion measured 2.5 × 1.7 cm and was surrounded by a hypointense rim on T2 sequences; it showed central T1 hypointensity and T2 hyperintensity, with no diffusion restriction in the core on apparent diffusion coefficient (ADC) maps. On FLAIR, the lesion displayed a heterogeneous “double ring” pattern, and after gadolinium administration both the peripheral and inner ring enhanced; the enhancing rims demonstrated diffusion restriction on ADC maps. Perfusion imaging showed no increased cerebral blood flow. Overall, these findings represented progression compared with prior imaging (Fig. 2).

#### Cerebrospinal fluid analysis

The patient was further investigated with lumbar puncture (normal cell count, elevated protein, cytology negative for malignancy, negative cultures, oligoclonal type II bands, indicative of local IgG production—IgG index = 0.823, normal values 0.28–0.66). Serum antibodies against MOG and anti-AQP4 were negative.

#### Differential diagnosis

The patient’s neurological symptoms raised several possible diagnoses. Primary central nervous system lymphoma was considered because of the underlying B-cell lymphoproliferative disorder. However, this diagnosis was considered unlikely based on the MRI findings, negative cerebrospinal fluid cytology, and the presence of cerebrospinal fluid-specific oligoclonal bands. Neurological involvement of Sjögren’s disease was also considered. Nevertheless, the clinical presentation, MRI findings, and cerebrospinal fluid results fulfilled both the original and revised McDonald criteria for MS, supporting the diagnosis of MS [28]. Neuromyelitis optica spectrum disorder and myelin oligodendrocyte glycoprotein antibody-associated disease (MOGAD) were excluded by negative anti-AQP4 and anti- MOG antibody tests.

Based on clinical, radiological, and cerebrospinal fluid findings, a diagnosis of tumefactive MS was established, and the patient was initiated on intravenous methylprednisolone pulse therapy (1g/day for three days).

#### Repeat gastric biopsy

**Fig. 3:**
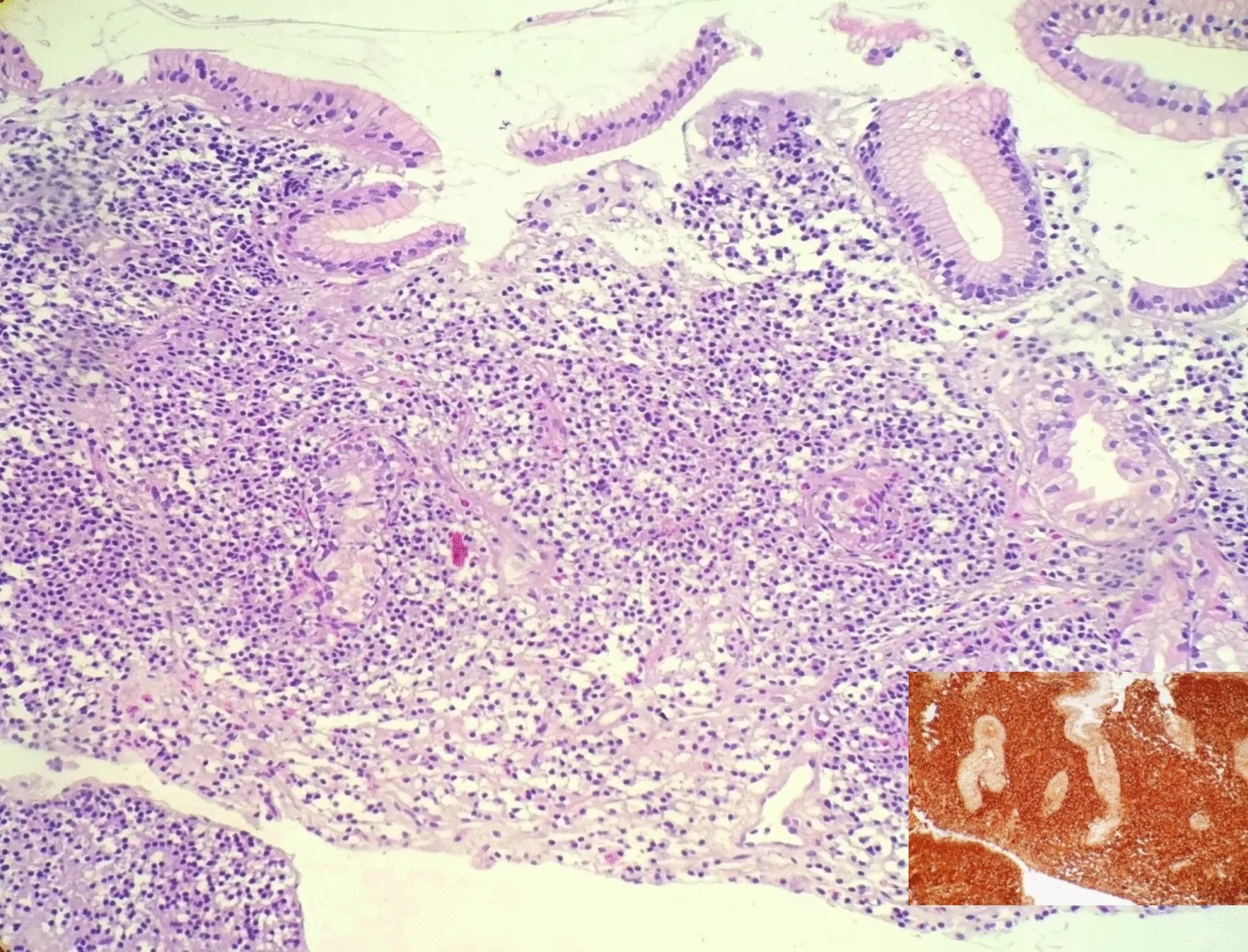
Gastric mucosa with dense lymphoplasmacytic infiltrates which destroy the gastric glands (Hematoxylin & Eosin stains, original magnification X100). Immunohistochemistry showed marked plasmacytic differentiation with lambda restricted positive cells (insert)

Nevertheless, in order to establish a proper therapeutic approach for the patient, it was necessary to confirm or exclude active MZL in the stomach. Therefore, a new gastroscopy was performed, nine months after the initial one, following eradication therapy for H. pylori, and new biopsies were obtained. Microscopic evaluation of the biopsy specimens revealed gastric mucosa with increased monoclonal CIg/λ⁺ plasma cell infiltration, consistent with MALT lymphoma exhibiting plasma cell differentiation. No microorganisms (e.g., H. pylori) or amyloid deposits were identified (Fig. 3).

### Therapeutic intervention

Based on these findings, the decision was made to initiate rituximab therapy (1 g on days 0 and 14).

### Follow-up and outcomes

**Fig. 4:**
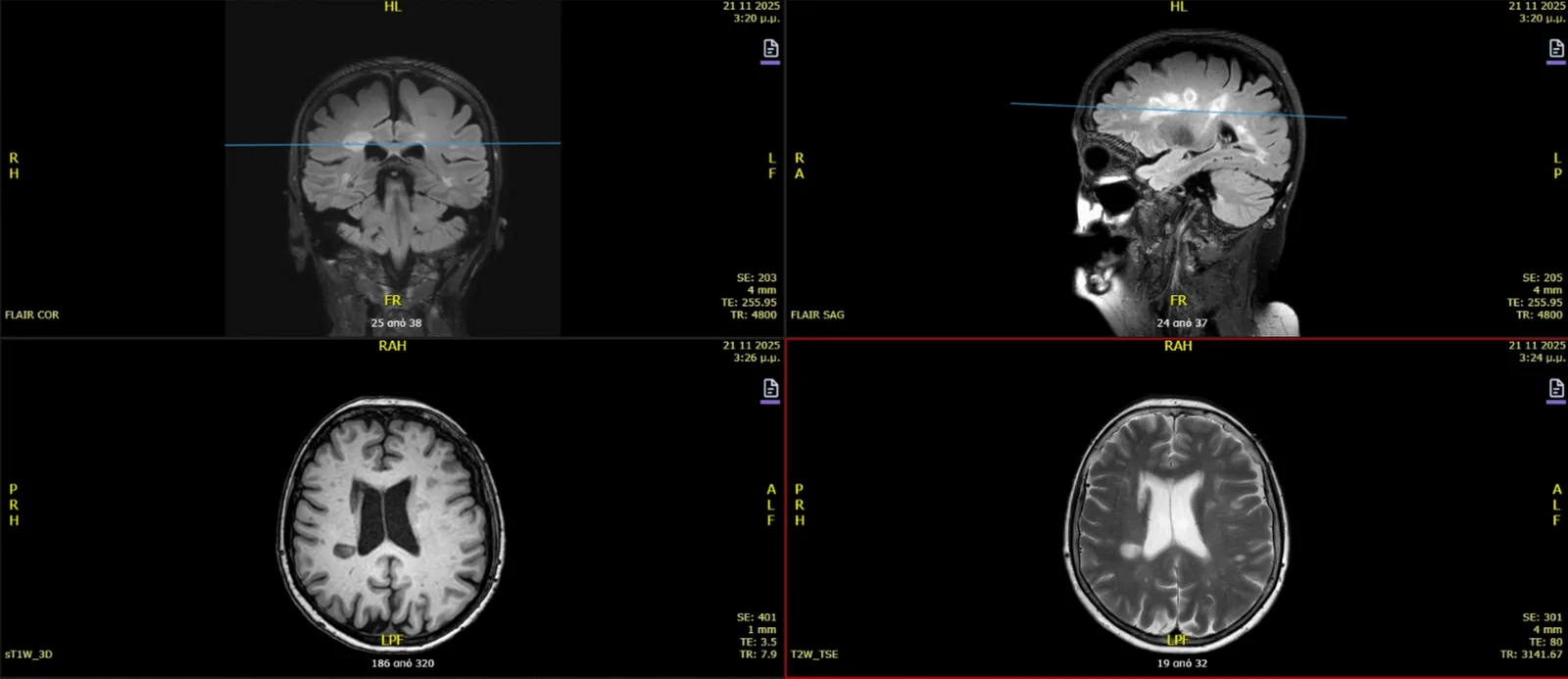
Repeat brain MRI scan of the patient – No new demyelinating lesions are revealed, while a substantial number of the pre-existing ones shows a marked reduction in size. Indicatively, the largest parietal lesion in the right hemisphere has a maximum diameter of 17 mm compared with 25 mm in the previous imaging

**Fig. 5:**
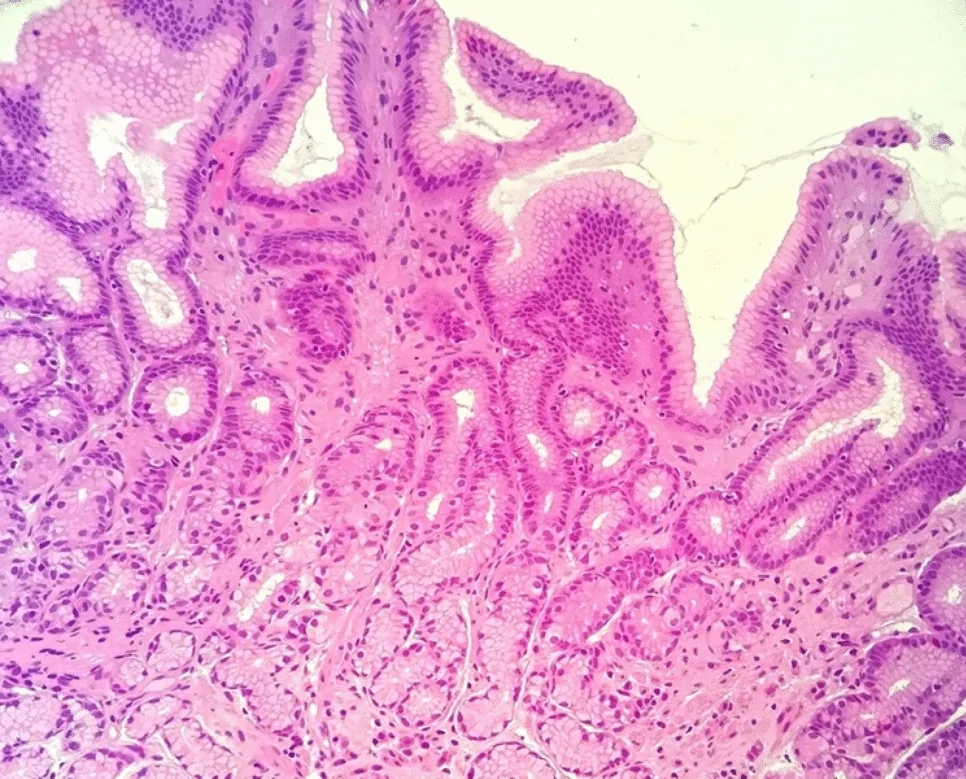
Gastric mucosa with scattered small lymphocytes and plasma cells, suggesting complete histological remission (Hematoxylin & Eosin stains, original magnification X200)

Six months later, the patient underwent a follow-up brain MRI, which did not reveal any new demyelinating lesions, while a substantial number of the pre-existing ones showed a marked reduction in size. Indicatively, the largest parietal lesion in the right hemisphere had a maximum diameter of 17 mm compared with 25 mm in the previous scan and was no longer surrounded by edema, while only its inner ring demonstrated contrast enhancement. Moreover, many of the other lesions no longer showed contrast enhancement (Fig. 4). A repeat gastroscopy with biopsy sampling was also conducted, revealing complete histological remission of the MALT lymphoma (Fig. 5).

At the six-month follow-up visit, the patient reported substantial clinical improvement. Her neurological symptoms, including gait instability and tremor, had significantly diminished, allowing her to regain functional independence in daily activities. Neurological examination revealed improved balance, with no new focal deficits. Gastrointestinal symptoms were absent, while her sicca symptoms remained stable without deterioration, and she continued receiving hydroxychloroquine therapy in addition to rituximab. Laboratory tests, including complete blood count, inflammatory markers, and routine biochemistry, revealed no significant abnormalities. The concurrent histological remission of MALT lymphoma, alongside the marked reduction in demyelinating lesion burden and neurological improvement, supported the efficacy of rituximab in targeting both lymphoproliferative and autoimmune components of this complex overlap syndrome.

A summary of the patient’s clinical course is presented in Supplementary Table 1.

## Methods

We conducted a systematic literature review to accompany this case report, following the PRISMA 2020 statement [29]. The review protocol was prospectively registered in PROSPERO (CRD4201332325).

A comprehensive literature search was performed in MEDLINE/PubMed, Scopus, the Cochrane Library, and the Directory of Open Access Journals (DOAJ) from database inception to July 2026. The search strategy combined terms related to Sjögren’s disease with terms for amyloidosis, gastric MALT lymphoma, and MS using database-specific syntax. The detailed search strategies for all databases are provided in Supplementary Data.

Two independent reviewers (N.Z. and G.D.) independently screened the titles and abstracts of all retrieved records according to the predefined eligibility criteria. Potentially eligible articles subsequently underwent full-text assessment. Any disagreements were resolved through discussion with a senior reviewer (P.V.), and consensus was reached for all inclusion decisions. Eligible studies included all publications, regardless of study design, reporting the coexistence of SjD, amyloidosis, gastric MALT lymphoma, and MS in the same patient. Articles published in languages other than English, French, or German and studies with unavailable full text were excluded. The study selection process is summarized in the PRISMA 2020 flow diagram (Supplementary Fig. 1).

As no published cases describing the complete coexistence of all four conditions were identified, supplementary targeted searches were subsequently performed to identify reports describing each possible combination of three of the four diseases (SjD–amyloidosis–MALT lymphoma, SjD–amyloidosis–MS, and SjD–MALT lymphoma–MS), in order to further assess the novelty of the present case.

For each eligible publication, data regarding study characteristics, patient demographics, clinical presentation, diagnostic findings, treatment, and outcomes were extracted. The literature review was conducted in accordance with the recently published CAse-BAsed REview sTandards (CABARET) recommendations [30].

## Results

We did not identify any published cases describing the coexistence of SjD, amyloidosis, gastric MALT lymphoma, and MS in the same patient. We therefore performed additional targeted searches for all possible combinations of three of the four conditions to better understand how this case relates to the existing literature. Only the combination of SjD, amyloidosis, and MALT lymphoma yielded relevant studies, whereas no publications were found for the combinations of SjD with amyloidosis and MS or SjD with MALT lymphoma and MS. We did not search for combinations of only two conditions because these associations have already been well described in previous studies.

### Combination of SjD, amyloidosis and MALT lymphoma

The supplementary search identified 15 eligible publications [31–44], including 14 single-patient case reports [31–44] and one retrospective case series [44] comprising eight patients, for a total of 22 patients. These studies were published between 1989 and 2026, with most (10/15, 66.7%) published after 2010. Three publications (20.0%) were available only as conference abstracts or posters rather than full peer-reviewed articles [32, 43, 44].

Most studies originated from Japan (6/15, 40.0%) [32, 35, 36, 38, 39, 44], followed by the United States (3/15, 20.0%) [34, 43, 45], the United Kingdom (2/15, 13.3%) [33, 37], France (2/15, 13.3%) [40, 42], Germany (1/15, 6.7%) [41], and Turkey (1/15, 6.7%) [31].

Overall, 21 of the 22 patients (95.5%) were female. All individually reported cases involved women, whereas the retrospective case series included seven women and one man. Patients ranged in age from 32 to 75 years, with ages of 34–73 years reported in the individual cases and a median age of 55 years in the case series. The time between the diagnosis of SjD and the development of amyloidosis and/or lymphoma ranged from simultaneous diagnosis to 25 years [31–45].

#### SjD characteristics

Serological findings were available for most of the individually reported cases. Anti-SSA/Ro antibodies were positive in 12 of the 14 patients (85.7%), whereas anti-SSB/La antibodies were reported in 6 patients (42.9%). A positive minor salivary gland biopsy (focus score or Chisholm grading) was documented in 7 of the 14 cases (50.0%). In the remaining cases, the biopsy was either not performed or not reported [31–45].

#### Amyloidosis characteristics

AL amyloidosis was confirmed in 21 of the 22 patients (95.5%), including all individually reported cases and seven of the eight patients in the retrospective case series. Among the 11 patients with available light-chain typing, κ-light chain restriction was found in 10 (90.9%), whereas λ-light chain restriction was reported in only one patient [31–45]. The light-chain subtype was not reported in three case reports [35, 41, 44].

Amyloid deposition was predominantly localized, with organ-confined disease observed in 12 of the 14 individual cases (85.7%). Systemic AL amyloidosis was reported in only two patient (39,40), while all patients included in the retrospective case series had localized pulmonary amyloidosis [31–38, 41–45].

The lung was the most frequently involved organ, accounting for 7 of the 14 individually reported cases. Salivary glands (parotid, labial, and/or submandibular) represented the second most common site, followed by the breast. After inclusion of the pulmonary case series, pulmonary involvement was observed in 15 of the 22 patients (68.2%), confirming the lung as the predominant site of amyloid deposition [31–45].

#### Lymphoma characteristics

MALT lymphoma was diagnosed in 13 of the 14 individual cases (92.9%), whereas one patient had lymphoplasmacytic lymphoma. Within the retrospective case series, MALT lymphoma was histologically confirmed in three of eight patients (37.5%). The remaining patients demonstrated localized pulmonary amyloid deposition, follicular bronchiolitis, or lymphoid hyperplasia without evidence of lymphoma. Overall, MALT lymphoma was confirmed in 16 of the 22 patients (72.7%) [31–45].

Helicobacter pylori (H. pylori) status was reported in only one study, in which upper gastrointestinal endoscopy was performed to exclude concomitant gastric MALT lymphoma, the patient tested negative for H. pylori. In all remaining studies, H. pylori status was either not reported or was not applicable because lymphoma involved non-gastric sites [31–45].

#### Treatment and outcomes

Treatment approaches varied across the reported cases. In two case reports, patients were referred to a hematologist [35, 40], but the subsequent treatment was not described. In one case, MALT lymphoma was diagnosed only at autopsy, consequently, no lymphoma-directed treatment was administered [32]. In one case report, the patient underwent surgery followed by adjuvant therapy, however, the type of adjuvant treatment was not specified [38]. Among the individually described patients, surgery with or without adjuvant chemotherapy or radiotherapy was the most common approach (5/19, 26.3%), followed by rituximab-containing regimens (3/18, 16.6%), watchful waiting (3/18, 16.6%), and chemotherapy and/or radiotherapy without rituximab (2/18, 11.1%). In the retrospective case series, only one patient received rituximab, whereas the remaining seven were managed conservatively [31–45].

Overall, clinical outcomes were favorable, with most patients achieving complete remission, durable disease control, or stable indolent disease during follow-up. Poor outcomes directly related to delayed diagnosis or disease progression were reported in only two individual cases: one patient was diagnosed only at autopsy [32], whereas another died from metastatic melanoma unrelated to SjD, amyloidosis, or lymphoma [41].

Importantly, none of the 15 included publications reported the coexistence of SjD, amyloidosis, lymphoma, and a concomitant central nervous system demyelinating disorder, including MS, further emphasizing the exceptional rarity of the present case [31–45].

## Discussion

In this study, we report the case of a woman with Sjögren’s disease (SjD) and multiple sclerosis (MS) who later developed pulmonary nodular amyloidosis and primary gastric MALT lymphoma. To determine whether similar cases had been reported, we performed a systematic literature review. We did not identify any published cases describing the coexistence of all four conditions. We therefore carried out additional targeted searches for all possible combinations of three of these conditions. Relevant studies were found only for the combination of SjD, amyloidosis, and lymphoma, whereas no reports were identified for the combinations of SjD with amyloidosis and MS or SjD with lymphoma and MS. We then reviewed the available evidence to better understand the relationship between SjD, amyloidosis, lymphoma, and MS, focusing on the mechanisms of lymphoma development in SjD and the possible immunopathogenic links between SjD and MS [31–45].

SjD is a chronic autoimmune disease lying at the intersection of autoimmunity and lymphomagenesis [46, 47]. It is marked by persistent B-cell hyperactivation and the formation of ectopic lymphoid structures, processes that may contribute to malignant lymphoid transformation. Among the complications of SjD, lymphoma development is regarded as the most severe [48]. In the context of our patient, the presence of nodular pulmonary amyloidosis further exemplifies the complex lymphoproliferative potential of SjD.

The current prevalent concept for lymphomagenesis related to SjD is built upon a slowly progressive and multistep process, centered around the B lymphocytes within the inflammatory lesion of the affected salivary glands (SGs). These B cells appear to evolve from a polyclonal population to an oligo- or monoclonal state, eventually progressing to malignancy. This transformation is thought to result from persistent antigenic stimulation combined with additional, yet undefined, microenvironmental and endogenous factors [3, 48].

MALT lymphomas develop at sites of persistent antigenic stimulation. Chronic B-cell activation is a hallmark of Sjögren’s disease and leads to the production of autoantibodies such as rheumatoid factor (RF), anti-Ro/SSA, and anti-La/SSB. Patients also commonly develop circulating immune complexes, hypergammaglobulinemia, oligoclonal B-cell expansion, and have an increased risk of B-cell non-Hodgkin lymphoma. From the early stages of the disease, patients may exhibit monoclonal immunoglobulins or light chains, while ineffective immunosurveillance against RF-producing B cells may promote clonal expansion [1].

**Fig. 6:**
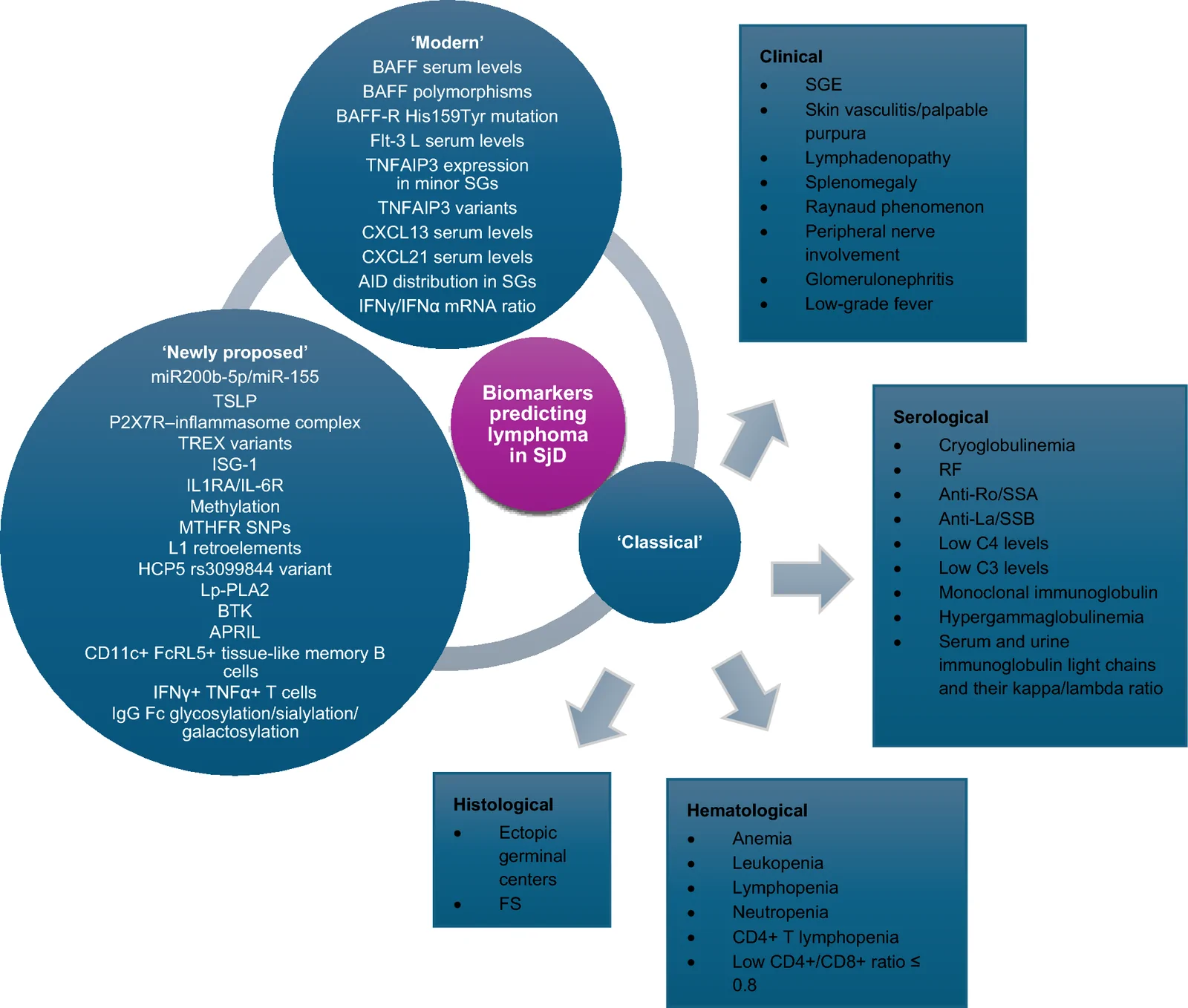
Summary of biomarkers predicting lymphoma in SjD [27, 31, 33, 34, 67]. *AID* activation-induced cytidine deaminase; *APRIL* A Proliferation-Inducing Ligand, *BAFF* B-cell activating factor, *BAFF–R* B-cell activating factor receptor, *CCL21*C-C Motif Chemokine Ligand 21, *BTK*: Bruton’s Tyrosine Kinase, *CXCL13* C-X-C motif chemokine ligand 13, *Flt-3 L* Fms-like tyrosine kinase 3 ligand, *FS* focus score, *HCP5* HLA complex P5, *IFN* interferon, *ISG-15* IFN-stimulated gene 15, *LILRA3* leukocyte immunoglobulin-like receptor A3, *Lp-PLA2* lipoprotein-associated phospholipase A2, *miR* microRNA, *MTHFR* methylene-tetrahydrofolate reductase, RF rheumatoid factor, SGE salivary gland enlargement, *TNFAIP3* tumor necrosis factor-alpha induced protein 3, *TNFα* Tumor Necrosis Factor-α, *TREX* Three-Prime Repair Exonuclease 1, *TSLP* Thymic Stromal Lymphopoietin, *SG* salivary gland

Clinically, salivary gland enlargement and cryoglobulinemia, often accompanied by vasculitis and hypocomplementemia, are among the strongest predictors of lymphoma in patients with SjD, reflecting advanced B-cell clonal expansion [46]. Several biomarkers have also been associated with lymphoproliferative complications, including BAFF, CXCL13, abnormalities of the interferon pathway, and regulators of NF-κB signaling [47, 49–51]. Figure 6 summarizes the main biomarkers associated with lymphoma development in Sjögren’s disease.

The mechanisms underlying the overlap between MS and Sjögren’s disease are not fully understood. Both conditions appear to share a complex interplay of genetic susceptibility and environmental triggers, particularly Epstein–Barr virus infection [52]. Both have been associated with HLA-DRB1 alleles and non-HLA immune-regulatory genes, including interferon regulatory factor 5 (IRF5), while genome-wide association studies have highlighted the JAK-STAT signaling pathway as a common pathogenic mechanism [53–55].

Both diseases are characterized by overactivation of T and B cells, abnormal cytokine signaling, and chronic immune stimulation. In Sjögren’s disease, activation of epithelial and dendritic cells leads to an interferon- and BAFF-driven immune response. In MS, antigen-presenting cells activate autoreactive T and B cells that attack myelin. These shared immune mechanisms suggest a common immunological background and may help explain why the two diseases can occur together through epitope spreading [10]. As suggested by Reske et al. [56], persistent B-cell activation may also lead to the formation of ectopic lymphoid structures in the central nervous system, similar to the germinal center-like structures seen in the salivary glands of patients with Sjögren’s disease.

The association between MS and lymphoma remains less well defined compared with the established relationship between lymphoma and SjD, as the available literature linking MS with MALT or extranodal MZL consists primarily of isolated case reports, lacking robust epidemiological studies that could establish a clear and generalizable association [57–59]. Nevertheless, published descriptions of cases of MALT lymphoma occurring in patients with MS do exist [60], suggesting a coexistence that is possible but uncommon, predominantly reported in individual cases rather than supported by large-scale data.

The patient’s neurological manifestations posed a differential diagnostic challenge. In this patient, we were convinced that there was a coexistence of two primary disorders, both MS and SjD. The patient met the McDonald criteria for MS, with distinct clinical episodes and typical lesions imaged by MRI, disseminated in space and time, and compatible CSF findings (oligoclonal type II bands) [26, 61]. Neuromyelitis optica spectrum disorders (NMOSD) were excluded since no positive anti-aquaporin-4 (anti–AQP4) and anti– Myelin Oligodendrocyte Glycoprotein (MOG) antibodies were detected. Finally, the temporal correlation between the onset of the two diseases and the significantly later onset of the demyelinating disease supports the hypothesis of the co-occurrence of two primary disorders.

MALT lymphoma most commonly arises in the stomach and is strongly associated with H. pylori infection. Eradication of H. pylori is therefore the standard first-line treatment and leads to remission in most patients. However, in H. pylori-negative cases, such as ours, other treatment options may be needed. Although eradication therapy can still be considered, response rates are generally lower than in H. pylori-positive disease [62]. Radiotherapy is highly effective for localized gastric MALT lymphoma, whereas rituximab-based chemoimmunotherapy is an effective option for patients who require systemic treatment [63, 64]. Endoscopic resection may be appropriate in selected patients with localized disease, while surgery is usually reserved for complications such as bleeding or perforation [65].

The treatment of SjD, on the other hand, seems to be a rather complex matter, since for over two decades, it has become a testing ground for modern therapies, with various drugs studied in clinical trials and still without any single regimen wholly accepted as an effective therapy for the disease to this day [63]. The patient has been receiving hydroxychloroquine, however in view of the MS and lymphoma diagnoses the initiation of rituximab was deemed appropriate, as it has been shown to be an effective treatment for both conditions, targeting both autoimmunity and lymphoproliferation [64, 66].

Following treatment, the patient achieved complete histological remission of the gastric MALT lymphoma, together with marked radiological improvement of the demyelinating lesions and significant neurological recovery. Although this outcome is encouraging, the contribution of rituximab cannot be determined from a single case, and further studies are needed to clarify its role in patients with overlapping autoimmune and lymphoproliferative disorders.

The present work also contributes to the scope of Rheumatology International by describing, to our knowledge, the first reported coexistence of SjD, pulmonary nodular amyloidosis, gastric MALT lymphoma, and MS. In addition to reporting this case, we performed a systematic literature review to better understand this rare combination of diseases. As no previous reports describing all four conditions were identified, we expanded our search to include all possible combinations of three of the four diseases. This allowed us to review the available evidence and better understand the relationship between these conditions. Together, the case and the literature review highlight the diagnostic challenges of these overlapping disorders and the importance of multidisciplinary management.

This study has several limitations. First, despite a comprehensive search across four databases, the available evidence was limited to a small number of case reports, one retrospective case series, and conference abstracts because of the rarity of this clinical association. Second, unpublished cases and reports from non-indexed sources may have been missed. Third, restricting the search to publications in English, French, and German may have resulted in the omission of relevant studies. Finally, our conclusions regarding possible shared immunopathogenic mechanisms are based on indirect evidence from studies evaluating combinations of three of the four diseases, as no published cases describing the coexistence of all four conditions were identified.

## Conclusion

To our knowledge, this is the first reported case of SjD, pulmonary nodular amyloidosis, gastric MALT lymphoma, and multiple sclerosis occurring in the same patient. This case highlights the diagnostic challenges associated with these overlapping disorders and the importance of multidisciplinary care. Although our patient had a favorable outcome following rituximab treatment, no conclusions can be drawn regarding its efficacy based on a single case. Further studies are needed to better understand the relationship between these conditions and to guide future diagnostic and therapeutic strategies.

## Supplementary Information

Below is the link to the electronic supplementary material.

Supplementary file1 (DOCX 52 KB)

## Data Availability

All data generated or analyzed during this study are included in this published article and its supplementary information files.

## Declarations

### Artificial intelligence use

No artificial intelligence tools were used in the preparation, writing, or analysis of this manuscript.
