# Supplementary material for: Pulmonary amyloidosis, gastric MALT lymphoma, and multiple sclerosis in a patient with Sjögren’s disease: a case report and review of the literature
Source: Rheumatol Int. 2026 Aug 25;46(9):244. doi: 10.1007/s00296-026-06276-0 (PMC13506517; doi:10.1007/s00296-026-06276-0)
Supplement: Supplementary file 1 [file 296_2026_6276_MOESM1_ESM.docx]

***Pulmonary Amyloidosis, Gastric MALT Lymphoma, and Multiple Sclerosis in a* Patient with Sjögren's Disease: A Case Report and Review of the Literature**

**Nikolaos Zintziovas^1^, Georgios A. Drosos^1,2^, Melina Yerolatsite^3^, Anna Goussia^4^, Vasiliki Kostadima^5^, Anastasia K. Zikou^6^, Paraskevi V. Voulgari^1^**

**Supplementary data**

| **Time** | **Clinical event** |
| --- | --- |
| **30 years before presentation** | Diagnosis of Sjögren's disease. |
| **3 years before presentation** | Ischemic stroke; pulmonary nodular amyloidosis diagnosed after lung nodule excision. |
| **9 months before presentation** | Initial gastroscopy suspicious for gastric MALT lymphoma; H. pylori eradication therapy; systemic amyloidosis excluded. |
| **Presentation** | Referral to the rheumatology clinic. |
| **Following presentation** | Progressive neurological deterioration; brain MRI showed increasing cerebral lesions. |
| **6 months later** | Brain MRI and CSF findings established the diagnosis of tumefactive multiple sclerosis. |
| **Shortly thereafter** | Repeat gastroscopy confirmed gastric MALT lymphoma; rituximab initiated. |
| **6-month follow-up** | Marked neurological improvement, regression of demyelinating lesions, and complete histological remission of MALT lymphoma. |

Supplementary Table 1: Timeline of the patient's clinical course.

**Search algorithm**

**PubMed**

("Sjogren Syndrome"[Mesh] OR "Sjögren syndrome"[tiab] OR "Sjogren syndrome"[tiab] OR "Sjögren's syndrome"[tiab] OR "Sjogren's syndrome"[tiab] OR "Sjögren disease"[tiab] OR "Sjogren disease"[tiab] OR "primary Sjögren syndrome"[tiab] OR "primary Sjogren syndrome"[tiab]) AND (("Amyloidosis"[Mesh] OR amyloidosis[tiab] OR amyloid[tiab] OR amyloidoma[tiab]) OR ("Lymphoma, Mucosa-Associated Lymphoid Tissue"[Mesh] OR "Lymphoma, B-Cell, Marginal Zone"[Mesh] OR "MALT lymphoma"[tiab] OR "mucosa-associated lymphoid tissue lymphoma"[tiab] OR "marginal zone lymphoma"[tiab] OR "extranodal marginal zone lymphoma"[tiab]) OR ("Multiple Sclerosis"[Mesh] OR "multiple sclerosis"[tiab] OR "tumefactive multiple sclerosis"[tiab]))

**Scopus**

TITLE-ABS-KEY ("Sjögren syndrome" OR "Sjogren syndrome" OR "Sjögren's syndrome" OR "Sjogren's syndrome" OR "Sjögren disease" OR "Sjogren disease" OR "primary Sjögren syndrome" OR "primary Sjogren syndrome") AND TITLE-ABS-KEY ((amyloidosis OR amyloid* OR "localized amyloidosis" OR "nodular amyloidosis" OR "pulmonary amyloidosis" OR "AL amyloidosis") OR ("MALT lymphoma" OR "gastric MALT lymphoma" OR "pulmonary MALT lymphoma" OR "mucosa-associated lymphoid tissue lymphoma" OR "marginal zone lymphoma" OR "extranodal marginal zone lymphoma") OR ("multiple sclerosis" OR "tumefactive multiple sclerosis" OR "tumefactive demyelinating lesion" OR "tumefactive demyelination"))

**Cochrane**

(Sjogren syndrome OR Sjögren syndrome OR Sjogren's syndrome OR Sjögren's syndrome OR Sjogren disease OR Sjögren disease OR primary Sjogren syndrome OR primary Sjögren syndrome) AND ((amyloidosis OR amyloid OR localized amyloidosis OR nodular amyloidosis OR pulmonary amyloidosis OR AL amyloidosis) OR (MALT lymphoma OR gastric MALT lymphoma OR pulmonary MALT lymphoma OR mucosa-associated lymphoid tissue lymphoma OR marginal zone lymphoma OR extranodal marginal zone lymphoma) OR (multiple sclerosis OR tumefactive multiple sclerosis OR tumefactive demyelinating lesion OR tumefactive demyelination))

**DOAJ**

(Sjogren disease OR Sjögren disease) AND (amyloidosis OR lymphoma OR multiple sclerosis)

**Identification of studies**

Records removed *before screening*:

Articles published in languages other than English, German, or French (n = 264)

Studies identified from (n=2966):

PubMed (n = 1118)

Scopus (n = 1756)

Cochrane (n=16)

DOAJ (n=76)

**Identification**

Studies screened (n=2702)

PubMed (n = 1014)

Scopus (n=1596)

Cochrane (n=16)

DOAJ (n=76)

Studies excluded

(n =2296)

**Screening**

Studies assessed for eligibility

(n = 406)

Studies excluded:

The 4 different diseases not assessed/combined in each case (n=406)

Studies included in review

(n = 0)

**Included**

***Supplementary Figure 1: PRISMA flow diagram of the study selection process.***

|  |  | **Amyloidosis** | | | **Lymphoma** | | **Treatment & Outcome** | | |
| --- | --- | --- | --- | --- | --- | --- | --- | --- | --- |
| **First Author** | **Year** | **Amyloidosis type (AL/AA/other)** | **Organ(s) involved** | **Localized vs systemic** | **Lymphoma type/subtype** | **Site** | **Treatment** | **Response/Outcome** | **Follow-up duration** |
| Taşkaraca Karabacak K (32) | 2025 | AL (kappa monoclonal) | Lung (bilateral, widespread) | Pulmonary | Extranodal marginal zone B-cell lymphoma | Parotid gland (initial, 7 yrs prior) → lung (current, multiple bilateral nodules) | R-CHOP planned (Rituximab, Cyclophosphamide, Doxorubicin, Vincristine, Prednisone) | NR (chemotherapy planned at time of publication; outcome not yet reported) | NR |
| Kisimoto W (33) | 2026 | AL (kappa-type) | Lung only | Localized (systemic spread excluded via bone marrow bx, cardiac MRI, GI eval, confirmed at autopsy) | MALT lymphoma, disseminated (diffuse plasma cell proliferation, lymphoepithelial lesions, κ light-chain restriction) | Gastric MALT lymphoma with involvement of the lungs, esophagogastric junction, urinary bladder, and mesenteric lymph nodes. | Prednisolone/mizoribine/azathioprine (SS/nephritis, earlier); tacrolimus (failed, pre-amyloidosis dx); melphalan + dexamethasone (pulmonary AL amyloidosis);No lymphoma-directed treatment; diagnosis made at autopsy. | Respiratory failure progressed despite therapy; MALT lymphoma undiagnosed until autopsy; death from intracerebral hemorrhage (dialysis-related, unrelated to amyloidosis/lymphoma) | 3 yrs (amyloidosis dx to death); lymphoma dx only at autopsy |
| Savage AWG (34) | 1989 | AL (lambda light chain type) | Parotid gland, gingiva, quadriceps muscle (multi-site) | Systemic/multi-site (parotid, gum, muscle); not confined to one organ | Lymphoplasmacytoid (lymphoplasmacytic) lymphoma, lambda-restricted | Parotid gland (primary); bone marrow (early involvement); thyroid tissue also showed excess lambda-restricted plasma cells | Chlorambucil 10 mg daily + local radiotherapy | Partial improvement in symptoms | NR |
| Dalal H (35) | 2023 | AL, peritumoral pattern | Pulmonary (ductal epithelium) | Localized (systemic involvement not reported) | Marginal zone B-cell lymphoma, MALT type, with light-chain restricted plasma cells (CD20+) | Breast (very rare site — <0.5% of all breast malignancies); confined to breast, no GI/other organ involvement | Left breast lumpectomy (surgical resection) | Negative margins; successful surgery; ongoing surveillance with screening mammography | NR specific duration (described as ongoing surveillance) |
| Murakami Y (36) | 2026 | AL kappa-type AND coexisting non-amyloid kappa light chain deposition disease | Lung (bilateral, multiple nodules + diffuse thin-walled cysts, lower lobe predominant) | Localized pulmonary | Pulmonary MALT lymphoma — CD20+ B lymphocytes (CD3-/CD5-/CD10-), lymphoepithelial lesions in bronchial epithelium, kappa light-chain restricted (RNA in situ hybridization) | Lung (peribronchiolar, forming lymphoepithelial lesions within bronchial epithelium) | No disease-specific treatment — conservative management with surveillance | Clinically and radiographically stable for ~1 year; no enlargement of nodules or cysts on serial imaging | ~1 year |
| Nakamura N (37) | 2002 | AL, kappa-type (amyloid deposits kappa+, AA-negative) | Lung — confined to the MALT lymphoma tumor stroma only, not present in other discrete sites | Localized AL κ amyloid deposition within the pulmonary MALT lymphoma | Pulmonary MALT lymphoma (stage I), CD20+, kappa light chain+, CD45RO-negative, lambda-negative; clonal rearrangement of immunoglobulin kappa light chain J region confirmed by Southern blot | Lung — left S6 tumor (38×38×20mm) | Surgical resection + combination chemotherapy (cyclophosphamide 1,100mg, hydroxydaunomycin/doxorubicin 75mg, oncovin/vincristine 2mg on day 1, prednisolone 100mg days 1–5, ×4 courses) | Irregular shadow in right lower lobe unchanged after chemotherapy; no recurrence for 16 months after tumor resection | 16 months |
| Odell EW (Case 1) (38) | 1998 | Localized amyloidosis (most likely AL) | Minor salivary glands — palatal mass (site of original MALT lymphoma) and a separate lower lip minor salivary gland (hyalinised nodule) | Localized (single minor salivary gland hyalinised/enlarged; no systemic amyloidosis identified) | Low-grade B-cell lymphoma of MALT type — palatal minor salivary gland; centrocyte-like cells, leukocyte common antigen+/L26+, UCHL1-/CD3-/MT1-negative | Palate (primary site) → separate lower lip minor salivary gland nodule (amyloid-containing) found 18 months after radiotherapy | Radiotherapy to posterior palate, 3600 cGy at 10 MeV in 18 fractions (fields did not include lips) | Free of lymphoma recurrence 3 years after radiotherapy; lip nodule (amyloid) excised, no further lesions apparent at 1–2 year follow-up | Recurrence-free 3 yrs (lymphoma); 1–2 yr follow-up after lip nodule excision |
| Ohara S (39) | 2019 | AL, kappa type-predominant (κ:λ ratio 20:1 by in situ hybridization) | Lung — multiple nodules (right S3, right S7, left S1+2, left S8) | Localized — nodular pulmonary amyloidosis (NPA), no tracheobronchial or alveolar septal involvement, asymptomatic | MALT lymphoma — CD20+/CD3+/CD138+/bcl-2+, kappa light-chain restricted (κ:λ 20:1), lymphoepithelial lesions (B-cell infiltration of bronchial epithelium) | Lung, multiple bilateral nodules; FDG-avid on PET | Wedge resection (left lower lobe nodule) + postoperative chemotherapy for MALT lymphoma (regimen/site not detailed — at another hospital) | Uneventful postoperative course, discharged POD4; further outcome/follow-up NR | NR |
| Watanabe Y (40) | 2024 | AL (presumed immunoglobulin light-chain origin per authors' discussion); specific kappa/lambda subtyping NOT reported in this case (unlike several others in this table) | Multi-site: left parotid gland (primary, resected), right sublingual gland, AND multiple anterior mediastinal masses | Extensive multi-site LOCALIZED disease (parotid + sublingual + mediastinal), NOT systemic | MALT lymphoma — CD20+, CD79a+, Bcl-2+, negative for Bcl-6/CD10/CyclinD1; lymphomatous component seen only at periphery of amyloid deposits (amyloid comprised most of the tumor mass) | Left parotid gland (primary, biopsy-confirmed) + right sublingual gland + anterior mediastinal masses (latter two presumed part of same disease spectrum by imaging/distribution, though lymphoma component only pathologically confirmed at the parotid site) | Left superficial parotidectomy (diagnostic/therapeutic); watchful waiting for remaining sublingual + mediastinal lesions (asymptomatic) | Remained asymptomatic without tumor progression over >1 year of watchful waiting | >1 year |
| Belfeki N (41) | 2019 | AL, kappa-type, SYSTEMIC (explicitly stated — confirmed at 2 separate sites: breast AND submandibular gland) | Breast (right upper quadrant) + submandibular gland (multi-site) | Systemic | Marginal zone B-cell lymphoma (MALT type) | Breast (primary presentation, right upper quadrant, 3cm mass) + submandibular gland + parotid gland enlargement on exam (multi-site/coexisting) | Referred to hematologist for specific treatment (regimen not detailed in this paper) | Diagnosis established; treatment outcome NR (pending hematology management at time of publication) | NR |
| Flaig MJ (42) | 2009 | Localized κ light-chain amyloid deposition (AL-type presumed). | Labial (lip) salivary glands — multiple submucosal nodules in lower lip and oral mucosa | Localized | Extranodal marginal zone B-cell lymphoma (MALT type) | Histologically confirmed in the labial salivary glands; additional salivary gland involvement (including parotids) was suspected clinically and radiologically but not biopsy-confirmed. | Chemotherapy indicated due to clinical relevance of IgM paraproteinemia + Bence-Jones proteinuria for already-impaired renal function: Bendamustine + Rituximab | Patient died 23 months later due to extensive metastasis from the malignant melanoma (UNRELATED to the MALT lymphoma/amyloidosis/SjD) | 23 months (death from unrelated melanoma metastasis) |
| Kambouchner M (43) | 2003 | Localized amyloidosis, probably AL (κ-associated). | Breast (left) — ductal/ductular localization | Localized — bone marrow and rectal mucosa biopsies both negative for systemic amyloidosis at 3.5-year follow-up | Low-grade marginal zone B-cell lymphoma (MZL, MALT-type) with plasmacytic differentiation | Breast (left, primary) + left axillary lymphadenopathy at presentation (axillary node itself was histologically unremarkable/reactive, no light chain restriction — Not lymphoma-involved) | No treatment given; watchful observation only | EXCELLENT outcome without any treatment: free of local MZL recurrence and visceral dissemination after 3.5 years of follow-up | 3.5 years without reccurence |
| Li H (44) | 2020 | AL, kappa-type | Lung (left apical nodule) | Pulmonary AL κ amyloidosis; systemic evaluation not reported. | Extranodal marginal zone lymphoma (MALT type) associated with IgA kappa immunoglobulin deposition | Lung — bilateral thin-walled cysts + irregular solid nodules; diagnosed via thoracoscopic lung biopsy | NONE — referred to hematology, annual surveillance only, no treatment given | Remained asymptomatic at last follow-up despite no treatment | At least 3 years; exact total follow-up NR |
| Miyazaki T (45) | 2018 | Pulmonary amyloidosis (light-chain restriction supporting AL-type) | Lung (bilateral multiple nodules) | Pulmonary amyloidosis; systemic evaluation not reported. | Extranodal marginal zone lymphoma (MALT type) | Lung (bilateral multiple nodules) + mediastinal mass (PET-avid, SUVmax 8.22) | Rituximab | PET scan showed nodules had disappeared (complete metabolic response); currently no organ disorders, symptomatic treatment only | NR exact duration; described as ongoing good response at time of poster |
| Baqir M (46) | 2013 | AL-kappa type in 7/8 | Lung (all 8) | Localized pulmonary (cystic + nodular disease, all 8) | MALT lymphoma in 3/8 patients-74F,48F, 52F (2 kappa-restricted, 1 lambda-restricted); remaining 5/8: amyloid only (3), follicular bronchiolitis (1), lymphoid hyperplasia (1) | Lung | Only 1/8 (symptomatic, extensive nodular/cystic disease) treated — rituximab; remaining 7/8 no specific therapy for amyloidosis/MALT | Indolent course overall: PFTs normal in 5/6 tested (mild impairment in 1); no nodule progressed to cyst even at longest follow-up; 6/8 cysts stable, 2/8 increased in size | Variable, up to 99 months |

Supplementary Table 2: Clinicopathological characteristics, treatment, and outcomes of published cases of Sjögren's disease associated with amyloidosis and lymphoma identified in the literature review.

Abbreviations: AL, amyloid light-chain; AA, amyloid A; bx, biopsy; CD, cluster of differentiation; CHOP, cyclophosphamide, doxorubicin, vincristine (Oncovin), and prednisone; EMZBCL, extranodal marginal zone B-cell lymphoma; FDG-PET, fluorodeoxyglucose positron emission tomography; GI, gastrointestinal; GI eval, gastrointestinal evaluation; IgA, immunoglobulin A; MALT, mucosa-associated lymphoid tissue; MRI, magnetic resonance imaging; NR, not reported; PET, positron emission tomography; PFT, pulmonary function test; POD, postoperative day; R-CHOP, rituximab, cyclophosphamide, doxorubicin, vincristine, and prednisone; RTX, rituximab; SS, Sjögren's syndrome; SUVmax, maximum standardized uptake value.
